# The integrity of synthetic magnesium silicate in charged compounds

**DOI:** 10.1038/s41598-021-02930-8

**Published:** 2021-12-09

**Authors:** Krystal L. House, Zhigang Hao, Yuxin Liu, Long Pan, Deirdre M. O’Carroll, Shiyou Xu

**Affiliations:** 1 Colgate-Palmolive Technology Center, 909 River Road, Piscataway, NJ 08854 USA; 2 Department of Chemistry and Chemical Biology, Rutgers University, 123 Bevier Road, Piscataway, NJ 08854 USA; 3 Department of Chemical and Biochemical Engineering, Rutgers University, 98 Brett Road, Piscataway, NJ 08854 USA; 4 Department of Materials Science and Engineering, Rutgers University, 607 Taylor Road, Piscataway, NJ 08854 USA

**Keywords:** Materials chemistry, Characterization and analytical techniques

## Abstract

Magnesium silicate is an inorganic compound used as an ingredient in product formulations for many different purposes. Since its compatibility with other components is critical for product quality and stability, it is essential to characterize the integrity of magnesium silicate in different solutions used for formulations. In this paper, we have determined the magnitude of dissociation of synthetic magnesium silicate in solution with positively charged, neutral, and negatively charged compounds using Scanning Electron Microscopy (SEM), Energy Dispersive X-ray Spectroscopy (EDS), and Liquid Chromatography-High Resolution Mass Spectrometry (LC-HRMS). The EDS results were verified through Monte Carlo simulations of electron-sample interactions. The compounds chosen for this study were positively charged cetylpyridinium chloride (CPC), neutral lauryl glucoside, and negatively charged sodium cocoyl glutamate and sodium cocoyl glycinate since these are common compounds used in personal care and oral care formulations. Negatively charged compounds significantly impacted magnesium silicate dissociation, resulting in physio-chemical separation between magnesium and silicate ions. In contrast, the positively charged compound had a minor effect on dissociation due to ion competition, and the neutral compound did not have such an impact on magnesium silicate dissociation. Further, when the magnesium ions are dissociated from the synthetic magnesium silicate, the morphology is changed accordingly, and the structural integrity of the synthetic magnesium silicate is damaged. The results provide scientific confidence and guidance for product development using synthetic magnesium silicate.

## Introduction

Synthetic magnesium silicates have broad applications in industry and are used extensively in pharmaceuticals, cosmetics, biodiesel purification, and chromatography. Laponite, a commercially available^1^ synthetic magnesium silicate, is used widely as a rheological modifier and filler for cosmetics and pharmaceuticals and has also been studied for its potential in biomedical applications, including 3D bioprinting and tissue regeneration^2–7^. In these fields, synthetic magnesium silicates also have potential in applications where natural magnesium silicates are used, including active ingredients or excipients for oral and topical pharmaceuticals and cosmetics^8–11^. The synthetic magnesium silicate, Magnesol, is used broadly in the biodiesel industry due to its adsorptive properties^12^. The active sites formed from the free silonal groups on the surface of magnesium silicate make it an excellent adsorbent^13^ resulting in extensive studies on its use as an adsorbent^13–16^, especially for used oils^15,17–21^ and wastewater treatment^22–28^. The adsorption properties are also exploited in chromatography, where synthetic magnesium silicate, sold as Florisil, is used widely in analytical and preparative chromatography applications^29–33^. Additional applications and studies of synthetic magnesium silicate include its use as a paint additive^34^, cement additive^35^, anticorrosion coating^36^, drug carrier in organic–inorganic hybrid materials^37^, and a polymer filler^16,38–49^. Due to the significant role of magnesium silicates in various industries, characterizing its stability in conjunction with other compounds and at different conditions for formulations is vital.

Synthetic and natural magnesium silicates have similar application potentials due to similarities in their structures. In the literature, there have been many studies on the synthesis of magnesium silicates^47,49–55^. Methods to control the physicochemical properties and morphology of magnesium silicates have also been examined, including the effects of surface modification^16,56–62^, the effects of additives during synthesis, including nonionic surfactants^16^, and sodium hydroxide^13^, as well as the effects of pH on synthesis^49^ and adsorption capacity^63^. Compared with the polycrystalline structure of natural magnesium silicate minerals, synthetic magnesium silicates commercially available are often amorphous and porous, potentially resulting in more significant dissociation in a liquid dispersion system. For example, in acidic solutions, Mg^2+^ ions can be leached out, i.e., dissociate from the silicate ion pairs, known as “acid leaching” of silicates^64^. Reaction of the free Mg^2+^ ions with other species in the solution can generate magnesium complexes that may alter product integrity and physical properties upon storage. Several studies have shown changes in magnesium silicate properties and morphology upon the addition of surfactants and ionic compounds^13,16,41^ or as an effect of pH^49,63^. However, to the best of our knowledge, studies on synthetic magnesium silicate stability under these conditions are lacking. Consequently, there is a need to determine the stability of synthetic magnesium silicate in various solutions and monitor possible changes in morphology.

Here, Scanning Electron Microscopy (SEM) and Energy Dispersive X-ray Spectroscopy (EDS) were used to determine the dissociation of synthetic magnesium silicate as a function of pH in positively charged, neutral, and negatively charged compounds. EDS has previously proven helpful for the study of magnesium silicate in the literature on scale inhibition in oil production^53,65^ and self-repairing lubricant technology^66^. The compounds chosen for the SEM/EDS study were positively charged cetylpyridinium chloride (CPC), neutral lauryl glucoside, and negatively charged sodium cocoyl glutamate since these compounds are often used in formulations for personal care or oral care products. The EDS results were verified through Monte Carlo simulations of electron-sample interactions using CASINO software^67^. Liquid Chromatography-High Resolution Mass Spectrometry (LC-HRMS) was used to verify and confirm the observed disassociation of Mg^2+^ ions from magnesium silicate. The results provide scientific confidence and guidance for product development using synthetic magnesium silicate and facilitate the use of SEM/EDS to characterize magnesium silicate integrity.

## Results

### SEM/EDS

**Figure 1:**
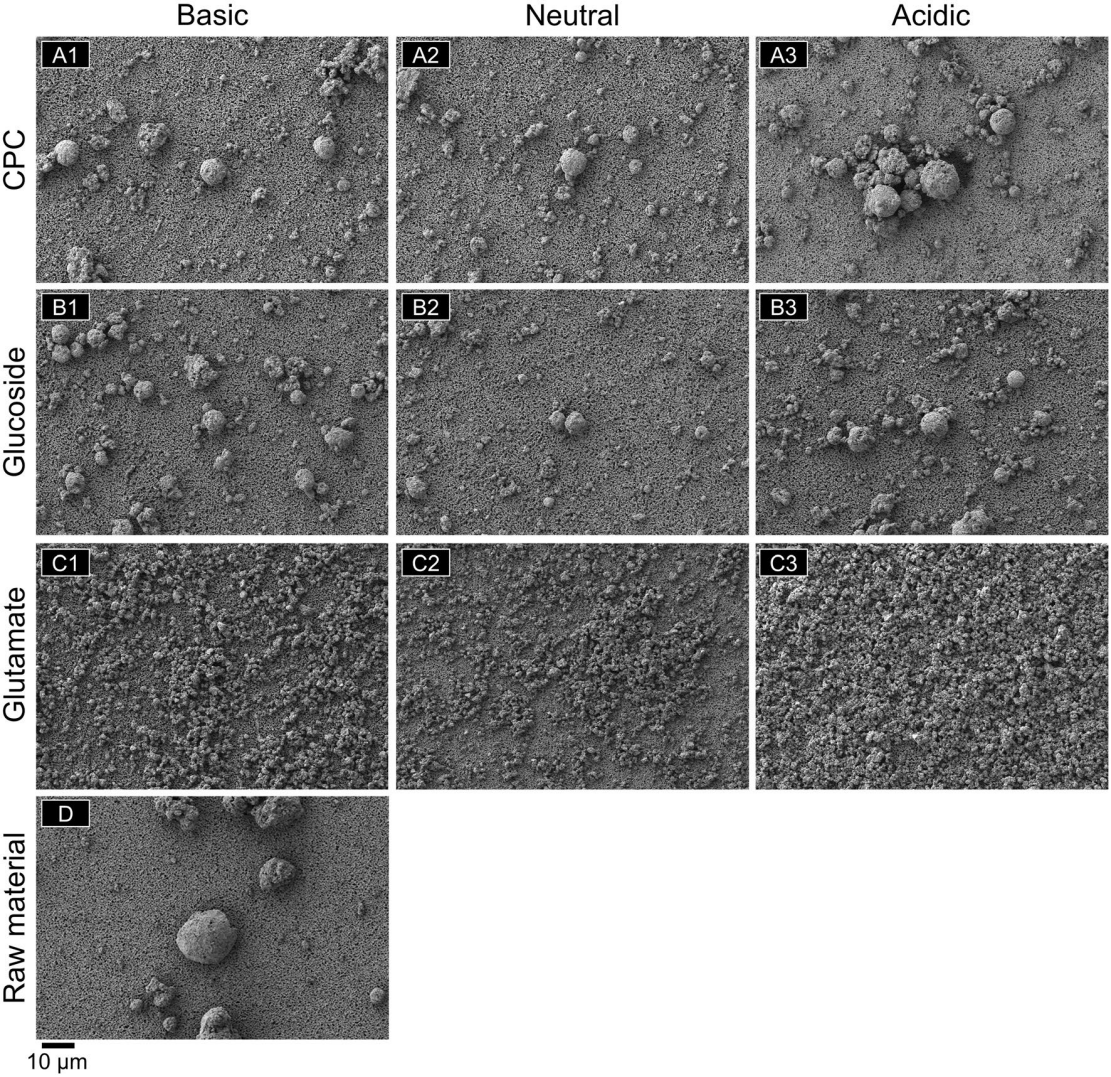
SEM images of collected solid, 1,000× magnification. Synthetic magnesium silicate dispersed in (**A1–3**) CPC, (**B1–3**) lauryl glucoside, and (**C1–3**) sodium cocoyl glutamate solutions and collected at (**A1**,** B1**, **C1**) basic, (**A2**, **B2**, **C2**) neutral, and (**A3**, **B3**, **C3**) acidic pH. (**D**) Original synthetic magnesium silicate raw material. Solid sample was collected from solution, dispersed in 2-propanol, deposited, and dried on a membrane filter used as a support substrate for imaging purposes.

To better understand the difference between the effect of positively charged, neutral, and negatively charged compounds on magnesium silicate dissociation, SEM/EDS analysis was conducted. The 3D structure of each compound is shown in Fig. S1 in the supplementary information**.** Representative 1000× magnification SEM images of the raw synthetic magnesium silicate particles (D) and the particles collected from each compound solution at each pH are shown in Fig. 1A1–C3. Table 1 in the Materials and Methods section shows the unique sample label assignment for each of the nine samples used for the SEM/EDS study. Images at additional magnifications of 500×, 5000×, 20,000×, and 50,000× can be found in the supplementary information in Figs. S2–S4.

**Table 1:** Unique labels for the samples prepared to study magnesium silicate dissociation as a function of pH.

	Basic	Neutral	Acidic
Cetylpyridinium chloride (Positively charged)	A1	A2	A3
Lauryl glucoside (Neutral)	B1	B2	B3
Sodium cocoyl glutamate (Negatively charged)	C1	C2	C3

The charge of each compound is listed in parenthesis.

From the SEM images in Fig. 1, all samples collected from positively charged CPC and neutral lauryl glutamate (A1–B3) consist of porous spheres, and the morphology of the raw material (D) was not changed after pH adjustment. However, samples collected from negatively charged sodium cocoyl glutamate solutions (C1-3) have been degraded as the images show many small particles. EDS spectra and mapping images were obtained to determine the elemental composition of the collected solid.

**Figure 2:**
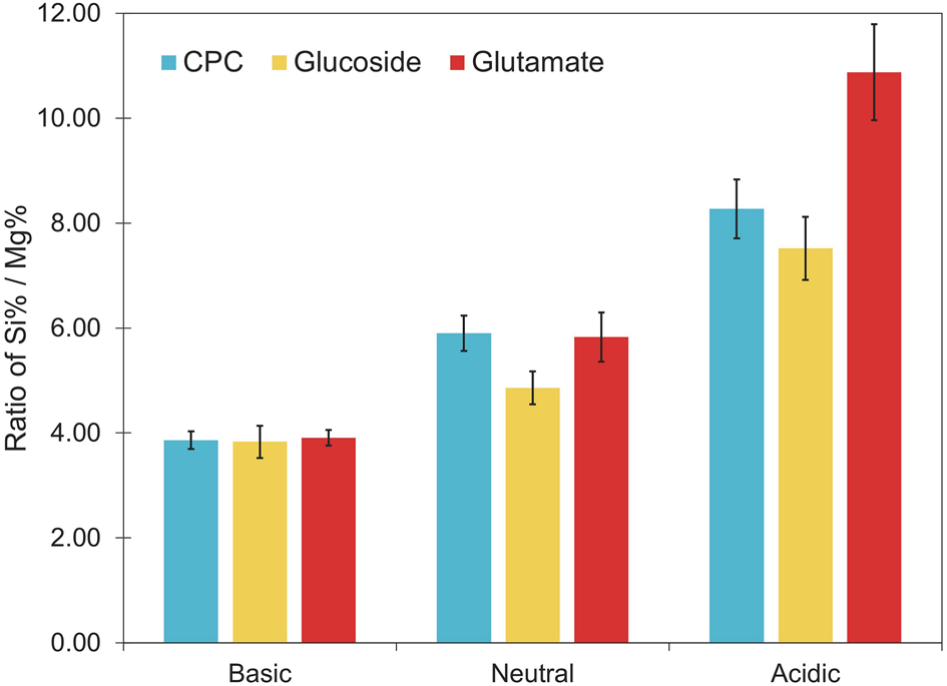
The ratio of percent Si over percent Mg in multiple point EDS spectra of synthetic magnesium silicate in basic, neutral, and acidic solutions of CPC, lauryl glucoside, and sodium cocoyl glutamate. Error bars are based on a minimum of 5 data points, with 13 data points being the average for all the samples. A full data table can be found in Table S1.

Point EDS spectra were measured for at least six particles for each of the nine samples. Only silicon (Si), magnesium (Mg), and oxygen (O) were included as elements of interest, and a ratio of the resultant percent Si over the percent Mg was calculated. A plot of the average ratios is shown in Fig. 2, where the ratio of percent Si/percent Mg increases when there is greater magnesium silicate dissociation. Representative spectra and a table of the calculated ratios can be found in Figs. S5–S13 and Table S1 in the supplementary information. As shown in Fig. 2, both pH and the charge of compounds affect Mg^2+^ ion dissociation.

**Figure 3:**
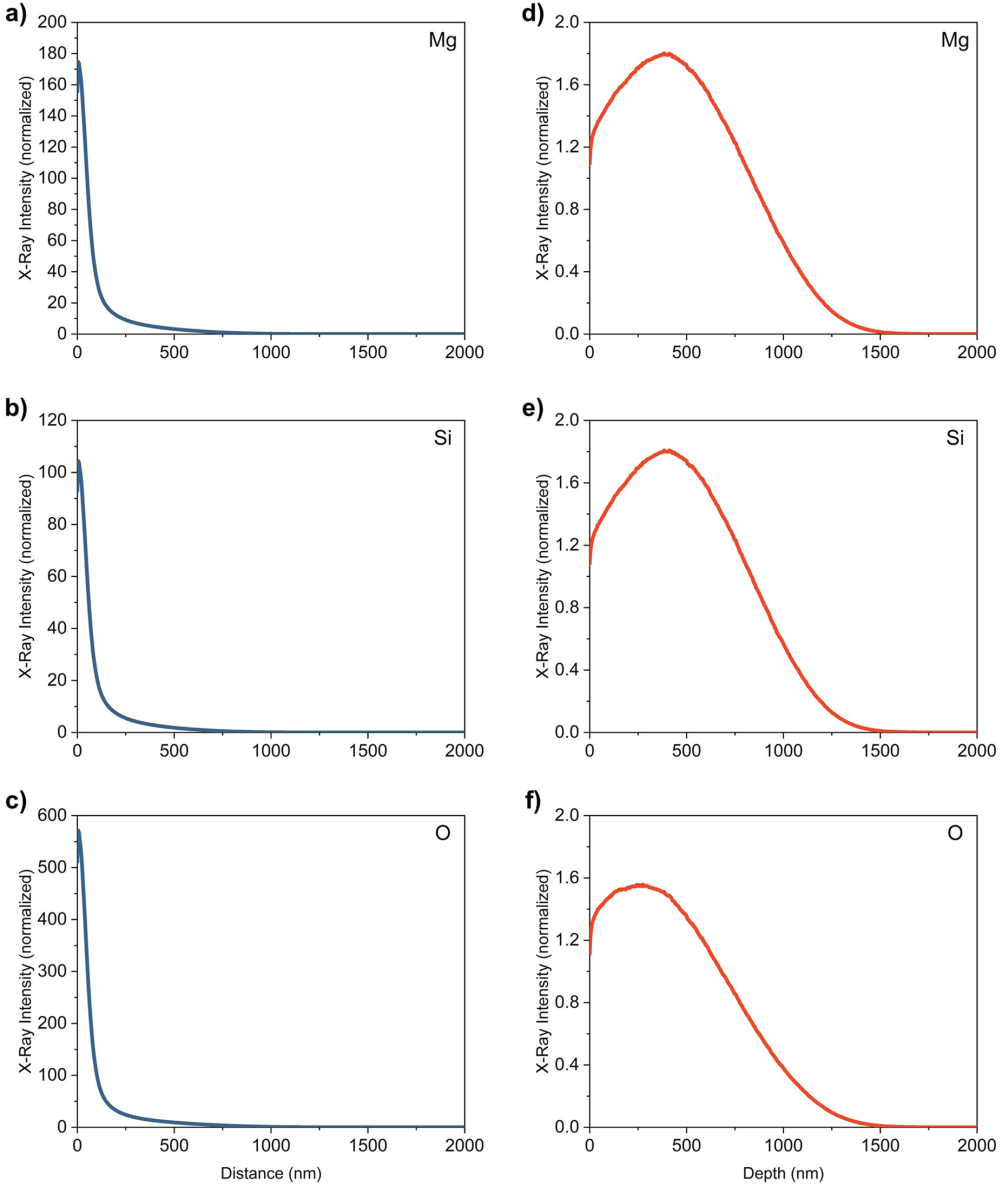
Generation of characteristic X-rays for 200,000 electrons targeting MgSiO at 10 keV with a beam radius of 125 nm. (**a**–**c**) Φ(*ρz*) curves for each element of a MgSiO sample. (**d**–**f**) X-ray intensities for each element as a function of radial distance (nm) from electron beam center. A MgSiO density (*ρ*) of 1.71896 g/cm^3^ and weight fractions of 0.1 for Mg, 0.39 for Si, and 0.51 for O determined from the typical Mg, Si, and O ratio measured in EDS point spectra of the neutral compound were used for the simulation.

To verify point EDS spectra were an accurate measurement of the solid particles, simulations of Monte Carlo electron-sample interactions were performed. CASINO software^67^ was used to simulate 200,000 electrons targeting MgSiO at 10 keV, with a beam radius of 125 nm. A MgSiO density (*ρ*) of 1.71896 g/cm^3^ and weight fractions of 0.1 for Mg, 0.39 for Si, and 0.51 for O determined from the typical Mg, Si, and O ratio measured in EDS point spectra of the neutral compound were used for the simulation. The software was used to generate characteristic X-rays for each element. The classical Φ(*ρz*) curves and radial distributions are shown in Fig. 3. Φ(*ρz*) is a relative generated intensity that varies with depth, first suggested by Castaing^68^ where the mass depth, *ρz*, is the product of the density of the sample, *ρ*, and the depth, *z*^69^. The Φ(*ρz*) curves give information on the X-ray generation depth of each element in the sample, while the radial distributions give lateral information. For each element, we can determine that the X-rays are only measured at a depth less than 700 nm into the sample and less than 1500 nm radially from the center of the beam. Since the typical particle size used for EDS point spectra was 6–10 μm, we can be positive that the results are obtained from the particle with no interference from the filter paper or substrate.

**Figure 4:**
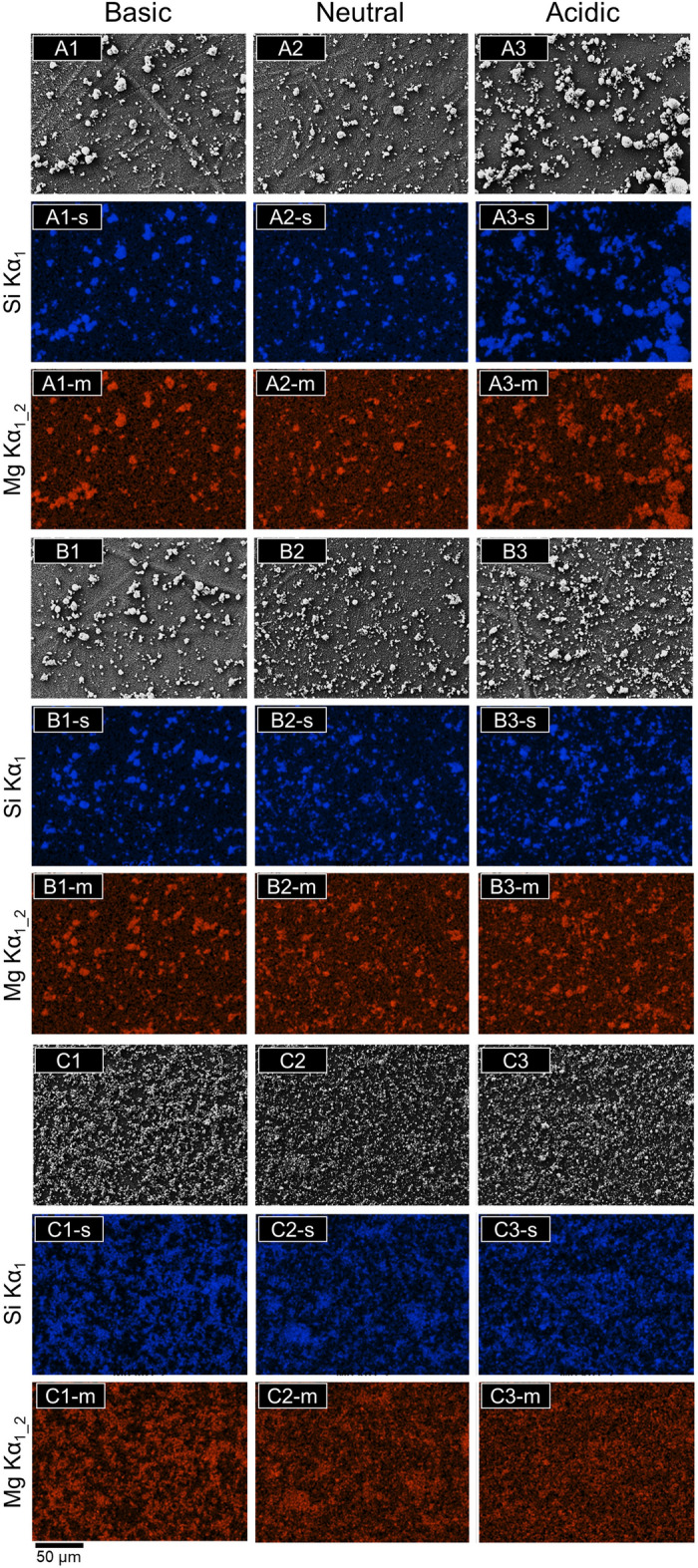
SEM images, Si (s), and Mg (m) EDS mapping images of synthetic magnesium silicate collected from (1) basic, (2) neutral, and (3) acidic (**A**) CPC solutions, (**B**) lauryl glucoside, and (**C**) sodium cocoyl glutamate solutions. After pH adjustment in solution, the solid sample was collected, dispersed in 2-propanol, deposited, and dried on a membrane filter used as a support substrate for imaging purposes.

From the EDS mapping results in Fig. 4, it is apparent that the amount of Mg decreases as the pH decreases while the amount of Si remains unchanged. This is seen in the color of the background around the particles. In the Si EDS mapping images, distinct particles are seen for all compound solutions at each pH. However, the Mg EDS mapping results show increasingly diffuse color in the background as pH decreases. This is especially apparent for the negatively charged sodium cocoyl glutamate solution at acidic pH. This indicates a decreasing Mg concentration in these samples. Overlap of Mg and Si in the other samples suggests that the elements are still combined in the magnesium silicate particle. We can conclude from these mapping images that the structural and chemical integrities of the original magnesium silicate are both adversely affected under low pH.

### LC-HRMS

To further understand the interaction from dissociated Mg^2+^ ions with charged compounds, simple solutions were prepared of magnesium chloride with two negatively charged compounds, sodium cocoyl glycinate and sodium cocoyl glutamate, as well as neutral lauryl glucoside separately. For both negatively charged compound solutions, a significant amount of precipitate was formed. No precipitate was formed in the lauryl glucoside solution. The differences are related to the charges. Lauryl glucoside is neutral and does not react with the positive Mg^2+^ ions. Therefore, no precipitate can be formed. In contrast, sodium cocoyl glycinate and sodium cocoyl glutamate are negatively charged compounds, and the interaction between the negative heads on the compounds and positive Mg^2+^ ions is the root cause for the precipitate formation. To confirm this hypothesis, the precipitates formed were analyzed by LC-HRMS.

**Figure 5:**
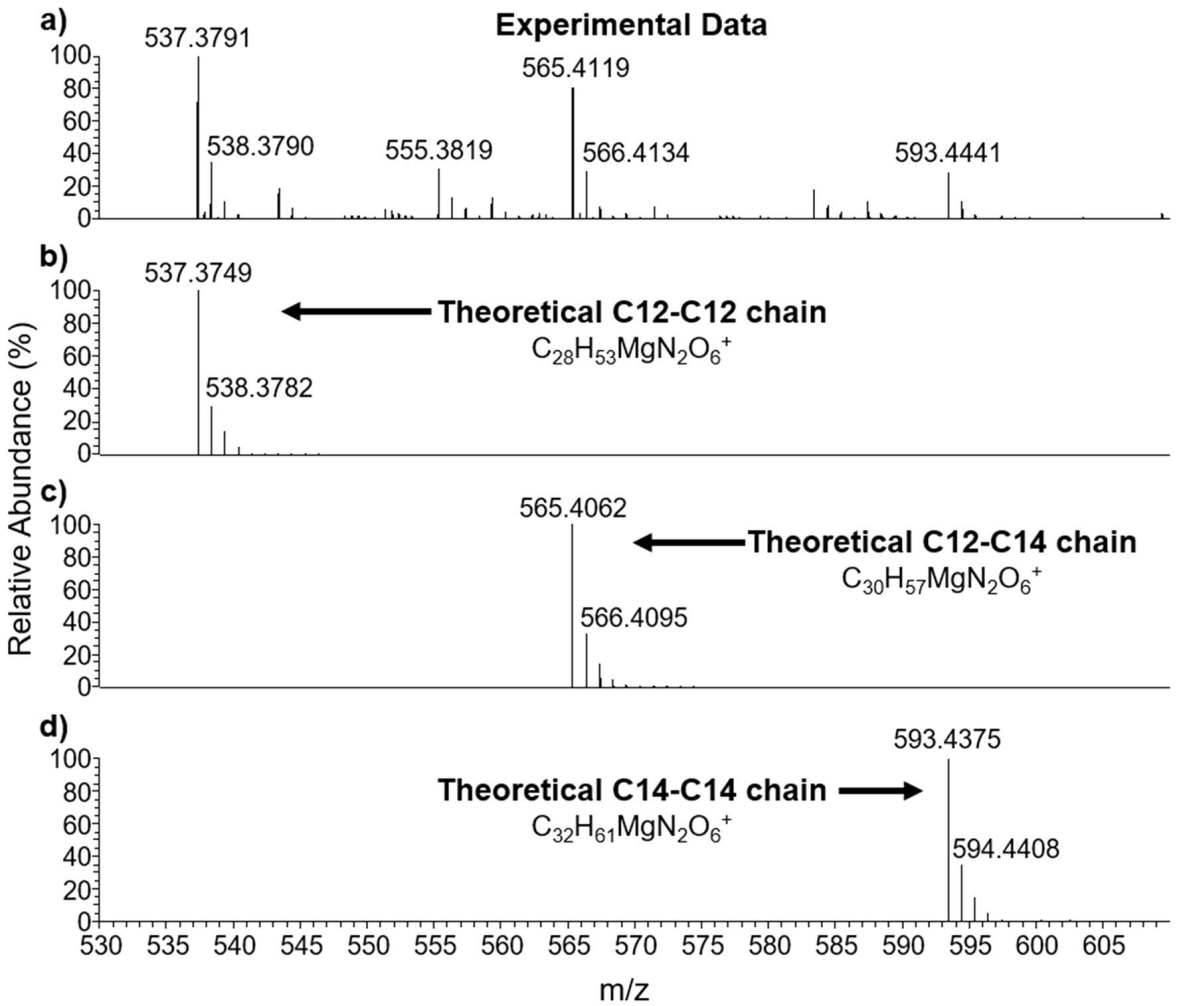
LC-HRMS results for the complex of magnesium-bis-cocoyl glycinate in methanol formed from magnesium chloride with sodium cocoyl glycinate: (**a**) experimental results, (**b**–**d**) theoretical results for complexes with varying chain lengths.

**Figure 6:**
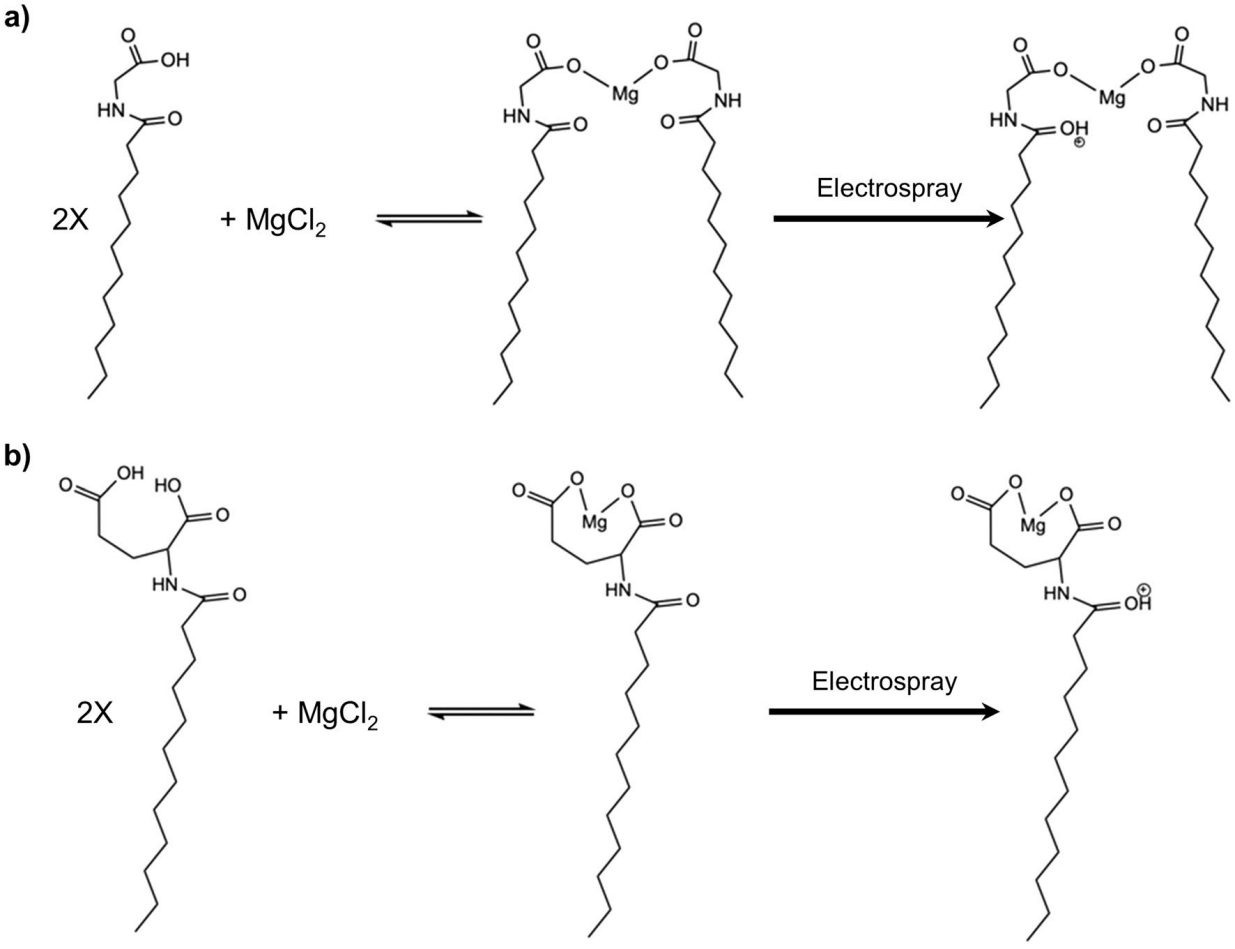
Schematic of the formation of magnesium complexes formed with negatively charged compounds. The chemical reactions for the formation of (**a**) magnesium-bis-cocoyl glycinate from magnesium chloride and sodium cocoyl glycinate and (**b**) magnesium-mono-cocoyl glutamate from magnesium chloride and sodium cocoyl glutamate and their positive charged forms in [M + H]^+^ LCMS detection mode (ChemDraw online version, https://perkinelmerinformatics.com/products/research/chemdraw/).

The precipitate was collected by filtration, dried, and redissolved into methanol. The methanol solution was analyzed by LC-HRMS. Figure 5 demonstrates the LC-HRMS results for the precipitate formed from magnesium chloride with sodium cocoyl glycinate. The precipitate formed can be explained by one mole of the positive Mg^2+^ ion chelated with two moles of negative ions from glycinate or glutamate functional groups at the compound head. The schematics of the formation of magnesium complexes formed with the negatively charged compound are shown in Fig. 6. The 3D structures of the complexes are shown in Fig. S1 e and f, respectively. The mixtures of magnesium chloride with lauryl glucoside and magnesium chloride with CPC were also analyzed by LC-HRMS. No Mg-compound complexes were found.

## Discussion

The SEM/EDS data illustrates that pH has the most significant effect on magnesium silicate dissociation. Since the ratio of percent Si/percent Mg increases as pH decreases for all compounds, the dissociation of synthetic magnesium silicate is greater at low pH. Using HCl to adjust the pH likely caused dissociation of magnesium silicate to form magnesium chloride (MgCl_2_), silicon dioxide (SiO_2_), and water according to Eq. (1) below^64^.

1 $${\text{MgSiO}}_{{3}} \left( {\text{s}} \right) \, + {\text{ 2HCl}}\left( {{\text{aq}}} \right) \, \to {\text{ MgCl}}_{{2}} \left( {{\text{aq}}} \right) \, + {\text{ SiO}}_{{2}} \left( {\text{s}} \right) \, + {\text{ H}}_{{2}} {\text{O}}\left( {{\text{aq}}} \right)$$

Once reacted in the low pH solutions, the highly water-soluble MgCl_2_ is removed in the washing steps while insoluble SiO_2_ remains, resulting in a lower elemental concentration of Mg in the EDS spectra of the particles.

Further, the negatively charged compound, sodium cocoyl glutamate, resulted in even greater dissociation, especially at acidic pH, than the neutral lauryl glucoside. The positively charged compound, CPC, also increased dissociation compared to the neutral compound due to competition between ions. We can conclude from these EDS point spectra that Mg^2+^ ions were leached from the original raw material as an effect of decreasing pH and with greater leaching facilitated by ionic compounds, particularly negatively charged compounds.

The LC-HRMS results indicate that the negatively charged compounds, which favor dissociation, will bond with the Mg^2+^ ions dissociated from synthetic magnesium silicate, supporting the structural change shown in the SEM/EDS results. Because the negatively charged compound facilitates the dissociation of the Mg^2+^ ions, the percent Si/percent Mg ratio of the solid in these solutions is higher, indicating more Mg^2+^ ions are leached out from the magnesium silicate particles, compared with positively charged and neutral compounds. Further, because Mg^2+^ ions are leached out, the structural integrity is destroyed, and the large spherical particles of the raw material deteriorate into small particles.

The structural changes may significantly affect the properties of synthetic magnesium silicate, so it may not meet its original purpose for use in various formulations. For example, if the porosity of synthetic magnesium silicate is the major targeted property, the structural change or the loss of structural integrity will significantly reduce its porosity. As a result, the adsorption property of magnesium silicate in the product will be reduced considerably, or the viscosity of the final product will be changed. Further, the Mg^2+^ ions leached from synthetic magnesium silicate will bond with the negatively charged compounds in the formulation or the final products, and insoluble particles or clusters can be formed. The insoluble particles may appear as big lumps in the final products, and the product quality may be challenged.

Thus, from the results obtained in this paper, during formulation or product development, attention should be paid to the pH level of the products and the selection of charged compounds when using synthetic magnesium silicate. A higher pH level will better preserve synthetic magnesium silicate. Further, positively charged or neutral compounds will not speed up the dissociation of synthetic magnesium silicate in low pH conditions. Thus, in a low pH formulation or product, the positively charged or neutral compounds are preferred.

## Conclusions

SEM, EDS, and LC–MS have been used to investigate the chemical and structural integrity of synthetic magnesium silicate as a function of pH and in solution with charged compounds. The results show that Mg^2+^ ions leach out from synthetic magnesium silicate at low pH conditions. Negatively charged compounds, such as sodium cocoyl glycinate or sodium cocoyl glutamate, significantly impact magnesium dissociation. These charged compounds react with Mg^2+^ ions leached from synthetic magnesium silicate, resulting in physio-chemical separation between the Mg^2+^ and silicate ions. Further, the reaction between Mg^2+^ ions and charged compounds increases the dissociation, and the structural integrity of the particles is destroyed. As a result, the large spherical particles are turned into small particles.

Therefore, for applications of synthetic magnesium silicate, which rely on its morphology and porosity, the pH level should be considered during the formulation process to avoid destroying the structural integrity of the spherical particles. Further, since Mg^2+^ ions are leached out from the particles at low pH, and they will bond with negatively charged compounds, it is essential to consider the possible phase separations of Mg^2+^ ions and the other compounds to avoid inhomogeneity in the final formulation and product. To obtain a more stable magnesium silicate compatible with negatively charged compounds, if low pH must be used in the formulation, further processing of the material is needed, such as thermal annealing, which can transform magnesium silicate from amorphous to crystalline states^64^. Further, if possible, neutral compounds should be selected to reduce the dissociation of Mg^2+^ ions and enhance the structural integrity of synthetic magnesium silicate; in other words, to better utilize the desired properties of magnesium silicate.

This research shows that it is critical to consider the pH value and the charge properties of the compounds used during the formulation of products using synthetic magnesium silicate to avoid possible issues caused by the dissociation of Mg^2+^ ions. Further, SEM/EDS is a straightforward method to provide essential information about the stability of different ingredients during the formulation process.

## Materials and methods

To prepare solutions for pH tests, three solutions of cetylpyridinium chloride (CPC, a positively charged compound, Sigma Aldrich, St. Louis, MO), lauryl glucoside (a neutral compound, BASF, Ludwigshafen, Germany), and sodium cocoyl glutamate (a negatively charged compound, BASF, Ludwigshafen, Germany) were prepared by dissolving 1 mol of each compound in 30 mL of deionized water. After the synthetic magnesium silicate (The Dallas Group of America, Inc, Whitehouse, NJ) was filtered (Whatman Grade 2 filter paper) and dried in a vacuum oven at 100 °C, 1 mol (based on anhydrous molar mass) was dispersed in each of the solutions with a stirring rate of 400 rpm for a minimum of 20 min at room temperature. Solid samples were collected from these solutions at the natural pH of each solution (approximately 10). The solid samples were collected after five series of centrifugation at 8000 rpm for 10 min. The supernatant was discarded, and the sample was redispersed in DI water between each centrifugation step. The pH of each solution was then adjusted with hydrochloric acid (Sigma Aldrich, St. Louis, MO, 36%) to approximately 7 and 5, with solid collected at each pH to obtain a total of nine solid samples. The unique labels for each of the nine samples are shown in Table 1. After the last centrifugation step, each sample was dispersed in 2-propanol, deposited and dried on a membrane filter, used as a support substrate for imaging, and analyzed using SEM/EDS as described.

Further, to determine the formation of magnesium complexes, a simple magnesium salt, magnesium chloride, was combined in solution with the charged compounds. First, 10% magnesium chloride (Sigma Aldrich, St. Louis, MO) and 10% lauryl glucoside were prepared individually in deionized water. The two solutions were randomly mixed with variable ratios, and no precipitation was observed. The pH ranged from 6 to 10. For the control experiment, solutions of 10% sodium cocoyl glycinate (a negatively charged compound, BASF, Ludwigshafen, Germany) and 10% sodium cocoyl glutamate were prepared in deionized water. Precipitation was observed when magnesium chloride solution was mixed with sodium cocoyl glycinate solution and sodium cocoyl glutamate solution separately. The precipitate was collected and redissolved in methanol solution and analyzed using LC-HRMS.

### SEM/EDS

A workstation (Crossbeam XB 540 FIB/SEM, Zeiss) was used to analyze the solid samples obtained at each pH. SEM imaging was optimized at an accelerating voltage of 2 kilovolts and a beam current of 200 picoamperes. EDS point spectra and mapping images were obtained at an accelerating voltage of 10 kilovolts and a beam current of 2 nanoamperes. SEM samples were prepared by dispersing each sample in 1.5 mL of 2-propanol and allowing 10 μL of the dispersion to dry on a membrane filter used as a support substrate for imaging purposes. The resultant solid samples on membrane filters were mounted and coated with gold/palladium through a sputtering process to reduce charging during SEM/EDS analysis.

### LC-HRMS

The precipitate made from magnesium chloride or synthetic magnesium silicate with negatively charged compounds was filtered and washed with water. The precipitate was dried and redissolved into methanol for LC-HRMS analysis with direct injection technology. The analysis was carried out with a Q-Exactive™ Orbitrap™ mass spectrometer (Thermo Scientific, San Jose, USA) equipped with a heated electrospray ionization source (HESI-II). Samples were analyzed in full-scan MS mode under positive polarity conditions using electrospray ionization. The compound and source parameters were optimized. The samples were introduced by direct injection and delivered by an HPLC mobile phase of 90% methanol–water. The optimized parameter settings were: sheath and auxiliary gas flow rate at 30 and 10 respectively, spray voltage 3.2 kV, capillary temperature 320 C, S-lens RF level 50, auxiliary gas heater temperature 400 C. Software used for operating the LC-HRMS instrument was Xcalibur™ (version4.1).

## Supplementary Information

Supplementary Information 1.

Supplementary Information 2.
