# Supplementary material for: The integrity of synthetic magnesium silicate in charged compounds
Source: Sci Rep. 2021 Dec 9;11:23717. doi: 10.1038/s41598-021-02930-8 (PMC8660837; doi:10.1038/s41598-021-02930-8)
Supplement: Supplementary file 1 — Supplementary Information 1. [file 41598_2021_2930_MOESM1_ESM.docx]

The Integrity of Synthetic Magnesium Silicate in Charged Compounds

Krystal L. House^1,2^, Zhigang Hao^1^, Yuxin Liu^1,3^, Long Pan^1^, Deirdre M. O’Carroll^2,4^, Shiyou Xu^1*^

1. Colgate-Palmolive Technology Center, 909 River Road, Piscataway, NJ 08854
2. Department of Chemistry and Chemical Biology, Rutgers University, 123 Bevier Road, Piscataway, NJ 08854
3. Department of Chemical and Biochemical Engineering, Rutgers University, 98 Brett Road, Piscataway, NJ 08854
4. Department of Materials Science and Engineering, Rutgers University, 607 Taylor Road, Piscataway, NJ 08854


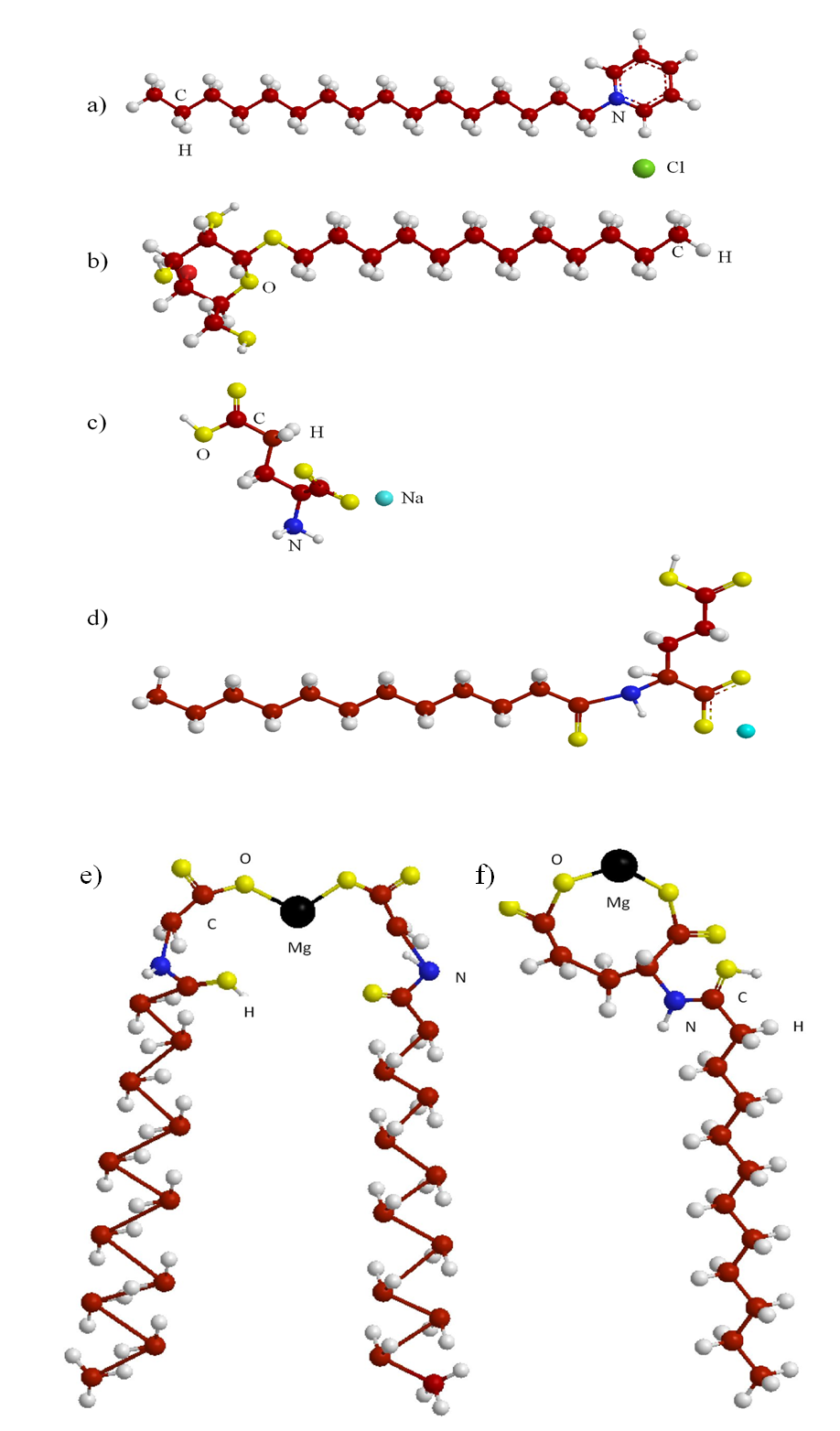


**Figure S1.** Molecular structure of the compounds: a) cetylpyridinium chloride (CPC), b) lauryl glucoside, c) sodium cocoyl glutamate, d) sodium cocoyl glycinate, and e) and f) Mg^2+^ and charged compound complexes (ChemDraw online version https://perkinelmerinformatics.com/products/research/chemdraw/)


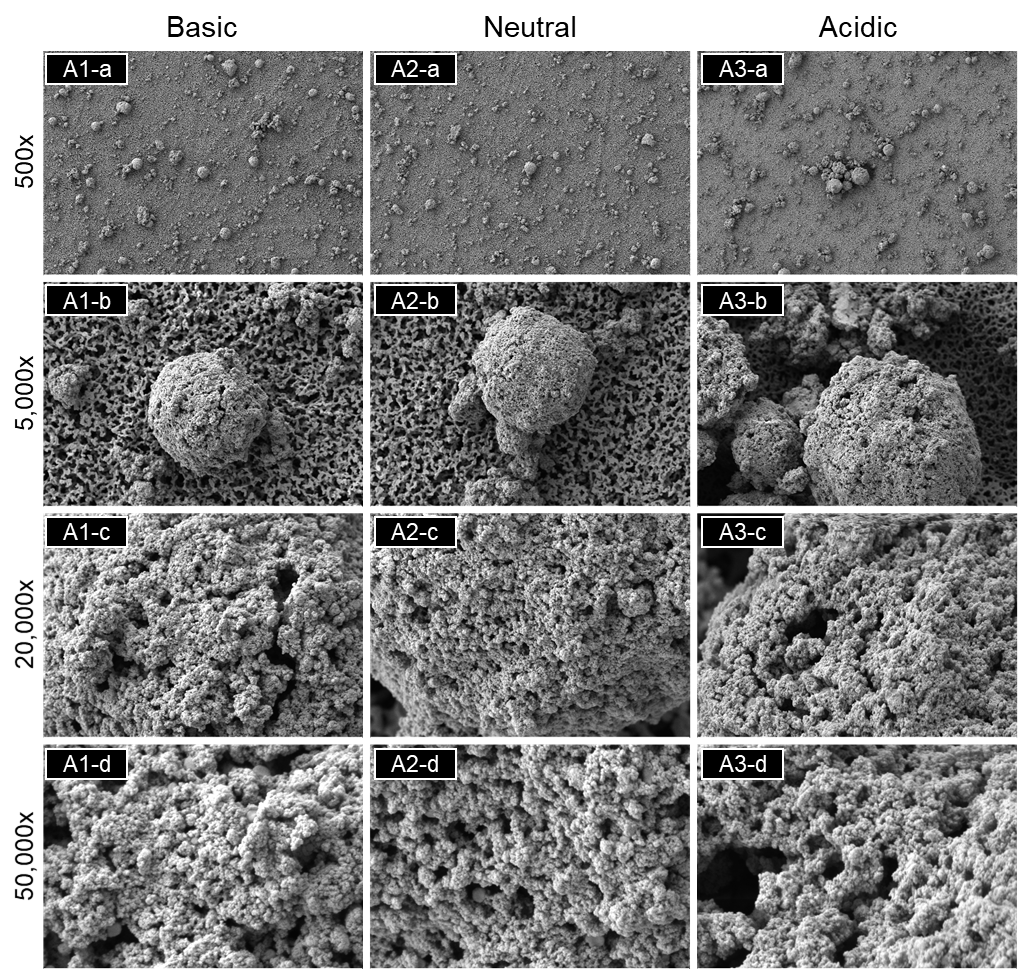


**Fig. S2** Additional magnification SEM images of synthetic magnesium silicate dispersed in CPC solutions and collected at A1-a-d) basic, A2-a-d) neutral, and A3-a-d) acidic pH. Magnifications are a) 500x, b) 5,000x, c) 20,000x, and d) 50,000x from top to bottom. After pH adjustment in solution, solid sample was collected, dispersed in 2-propanol, deposited, and dried on a membrane filter used as a support substrate for imaging purposes

**
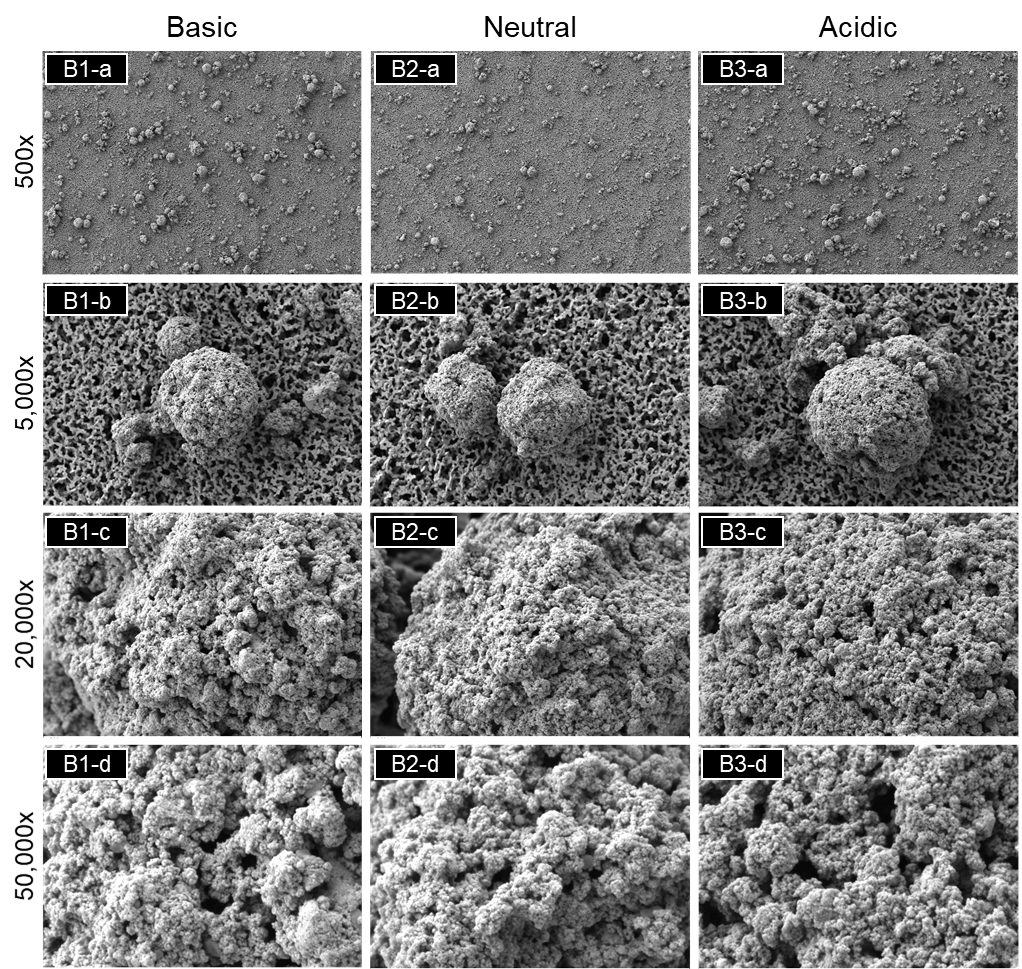
**

**Fig. S3** Additional magnification SEM images of synthetic magnesium silicate dispersed in lauryl glucoside solutions and collected at B1-a-d) basic, B2-a-d) neutral, and B3-a-d) acidic pH. Magnifications are a) 500x, b) 5,000x, c) 20,000x, and d) 50,000x from top to bottom. After pH adjustment in solution, solid sample was collected, dispersed in 2-propanol, deposited, and dried on a membrane filter used as a support substrate for imaging purposes

**
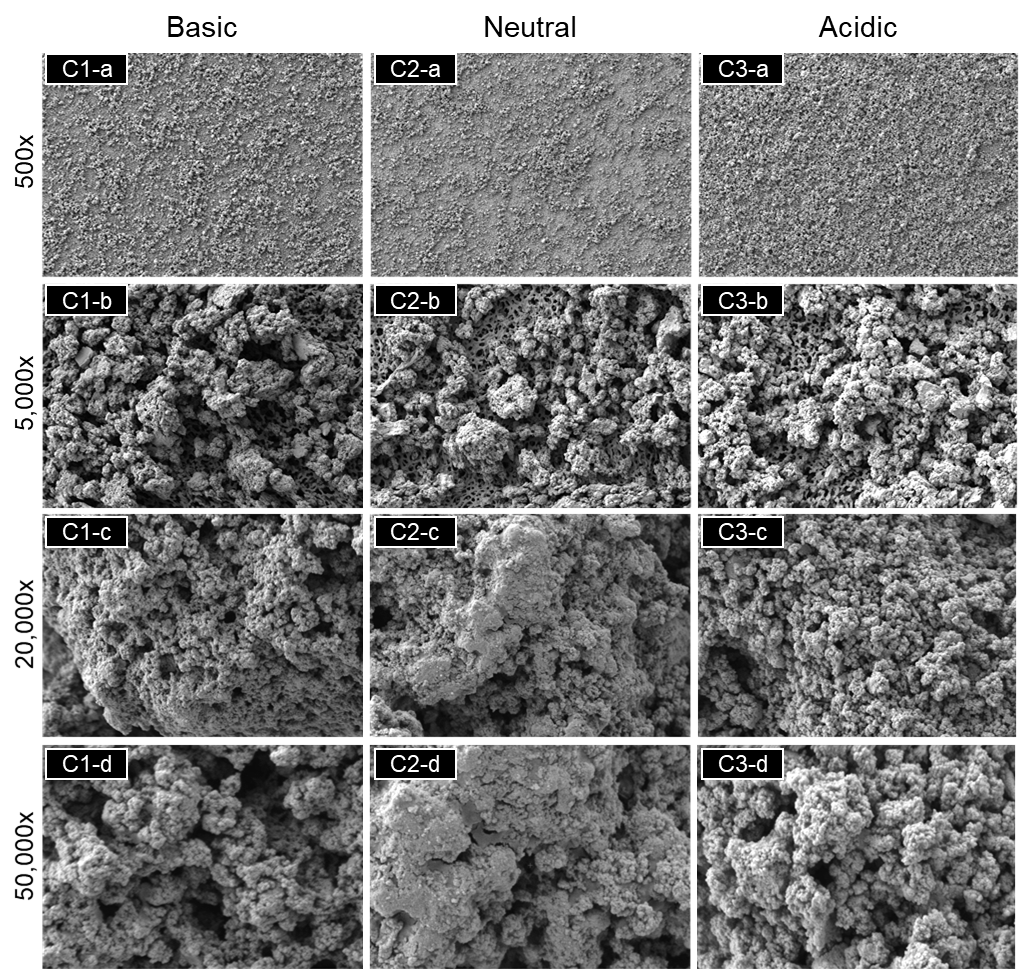
**

**Fig. S4** Additional magnification SEM images of synthetic magnesium silicate dispersed in sodium cocoyl glutamate solutions and collected at C1-a-d) basic, C2-a-d) neutral, and C3-a-d) acidic pH. Magnifications are a) 500x, b) 5,000x, c) 20,000x, and d) 50,000x from top to bottom. After pH adjustment in solution, solid sample was collected, dispersed in 2-propanol, deposited, and dried on a membrane filter used as a support substrate for imaging purposes


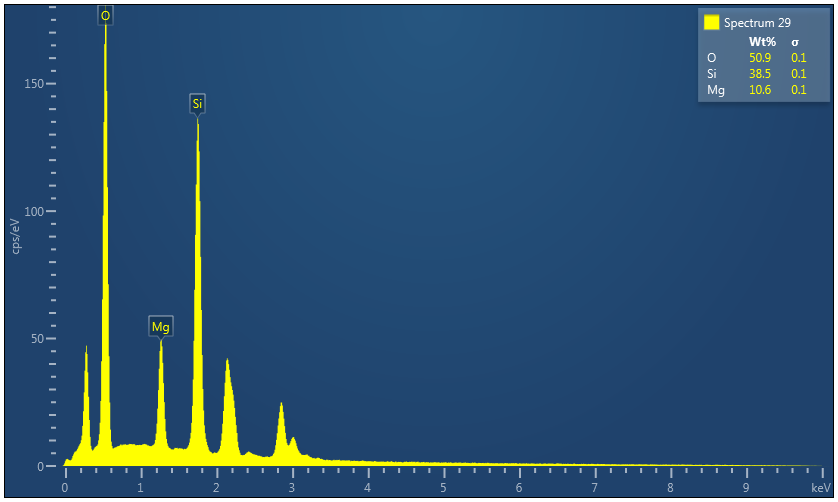


**Fig. S5** Representative point EDS spectra of synthetic magnesium silicate in basic solution of CPC


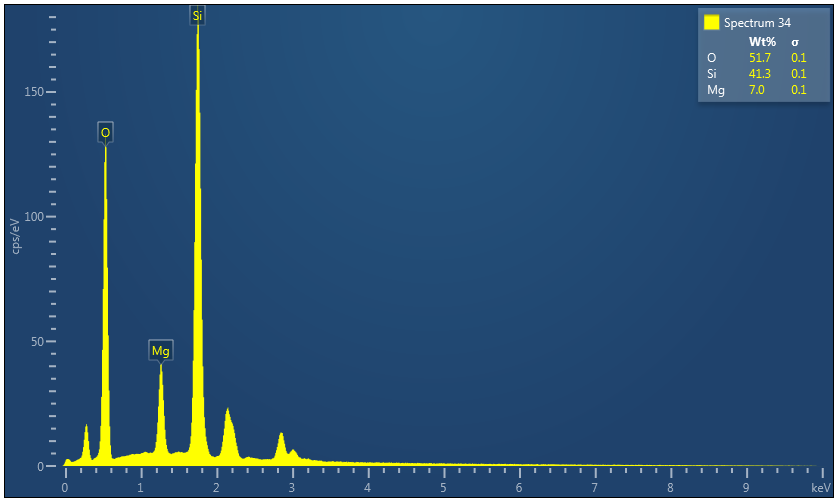


**Fig. S6** Representative point EDS spectra of synthetic magnesium silicate in neutral solution of CPC


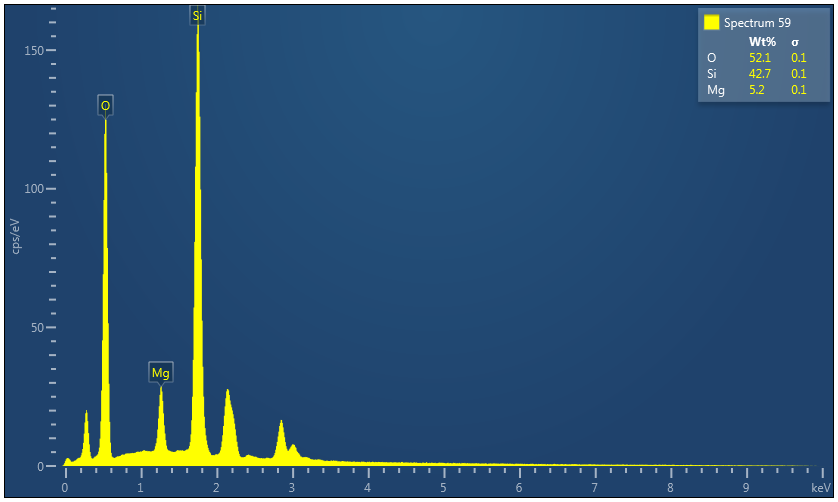


**Fig. S7** Representative point EDS spectra of synthetic magnesium silicate in acidic solution of CPC


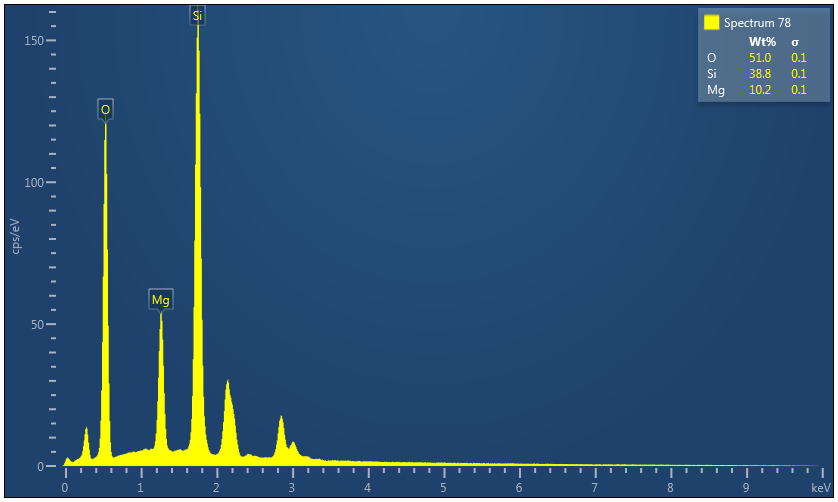


**Fig. S8** Representative point EDS spectra of synthetic magnesium silicate in basic solution of lauryl glucoside


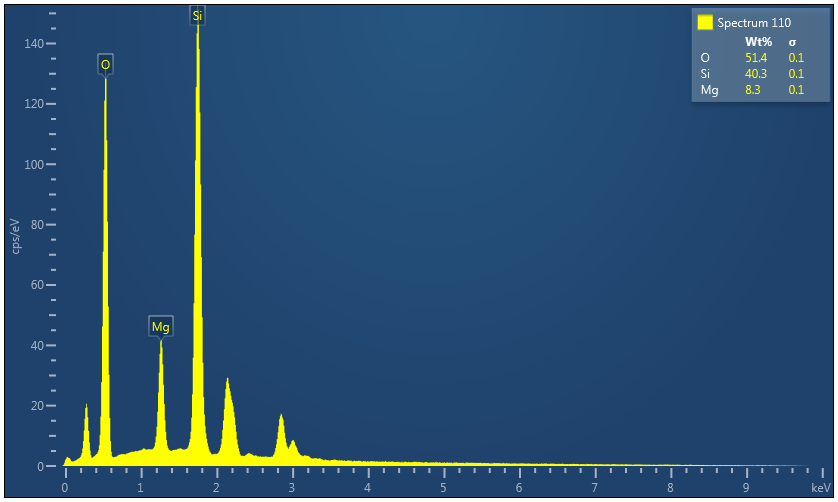


**Fig. S9** Representative point EDS spectra of synthetic magnesium silicate in neutral solution of lauryl glucoside


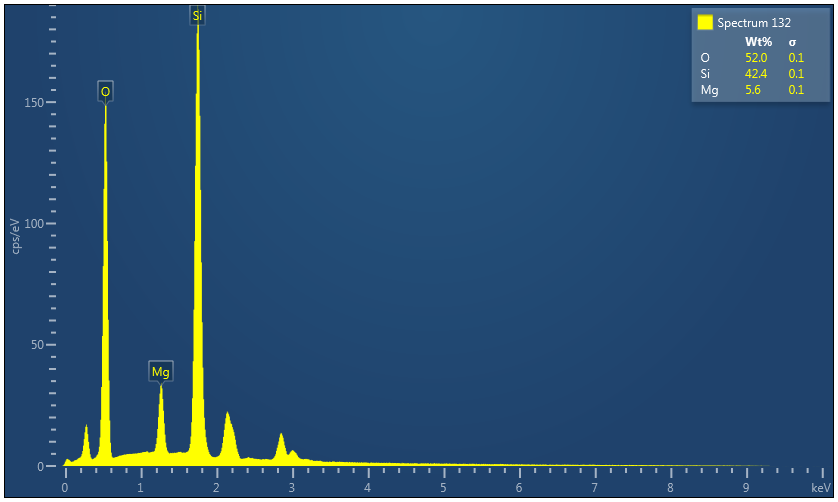


**Fig. S10** Representative point EDS spectra of synthetic magnesium silicate in acidic solution of lauryl glucoside


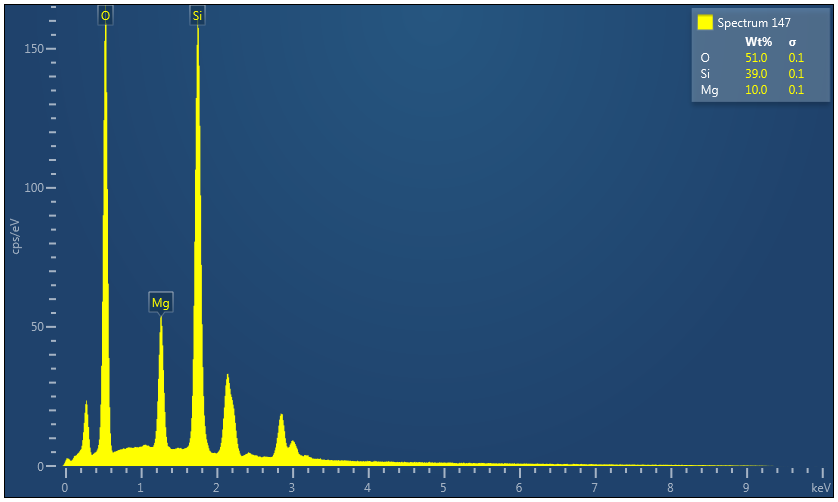


**Fig. S11** Representative point EDS spectra of synthetic magnesium silicate in basic solution of sodium cocoyl glutamate


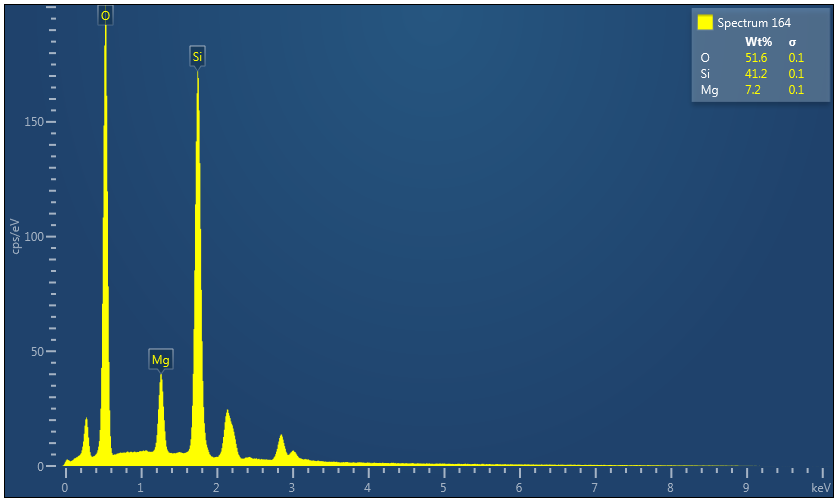


**Fig. S12** Representative point EDS spectra of synthetic magnesium silicate in neutral solution sodium cocoyl glutamate.


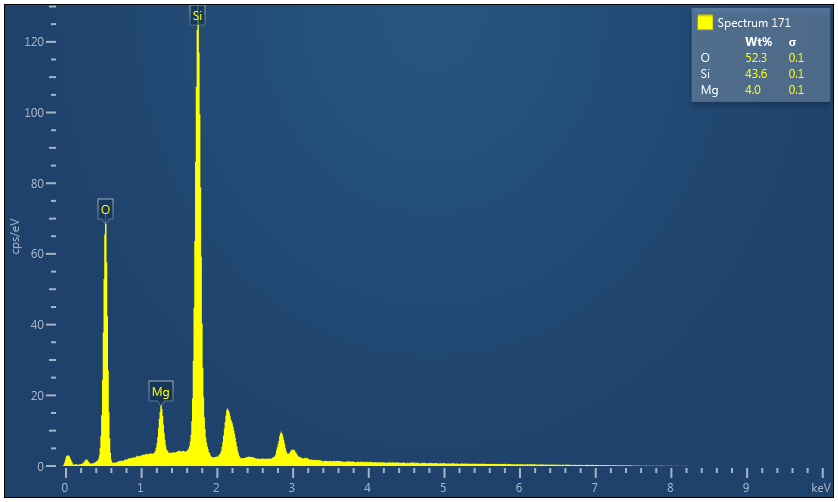


**Fig. S13** Representative point EDS spectra of synthetic magnesium silicate in acidic solution sodium cocoyl glutamate

**Table S1** Ratio of Si percent over Mg percent in multiple-point EDS spectra of synthetic magnesium silicate in basic, neutral, and acidic solutions of CPC, lauryl glucoside, and sodium cocoyl glutamate

|  | **Acidic Solution** | **Neutral Solution** | **Basic Solution** |
| --- | --- | --- | --- |
| **CPC** | 7.73 | 5.54 | 3.64 |
|  | 7.73 | 5.54 | 3.68 |
|  | 7.73 | 5.54 | 3.95 |
|  | 7.73 | 5.72 | 4.00 |
|  | 7.87 | 5.82 | 4.04 |
|  | 7.87 | 5.90 |  |
|  | 7.89 | 5.90 |  |
|  | 8.04 | 5.90 |  |
|  | 8.04 | 6.00 |  |
|  | 8.04 | 6.53 |  |
|  | 8.04 | 6.53 |  |
|  | 8.21 |  |  |
|  | 8.39 |  |  |
|  | 8.56 |  |  |
|  | 8.58 |  |  |
|  | 8.58 |  |  |
|  | 9.17 |  |  |
|  | 9.39 |  |  |
|  | 9.60 |  |  |
| **Glucoside** | 6.77 | 4.36 | 3.95 |
|  | 6.89 | 4.36 | 4.04 |
|  | 6.89 | 4.48 | 3.94 |
|  | 6.89 | 4.60 | 3.80 |
|  | 7.02 | 4.60 | 4.36 |
|  | 7.02 | 4.73 | 3.81 |
|  | 7.44 | 4.79 | 3.99 |
|  | 7.57 | 4.86 | 3.99 |
|  | 7.57 | 5.08 | 3.76 |
|  | 7.73 | 5.14 | 3.40 |
|  | 7.73 | 5.14 | 3.90 |
|  | 7.89 | 5.14 | 3.77 |
|  | 8.04 | 5.22 | 3.68 |
|  | 8.58 | 5.22 | 4.65 |
|  | 8.76 | 5.22 | 3.60 |
|  |  |  | 3.63 |
|  |  |  | 3.60 |
|  |  |  | 3.25 |
|  |  |  | 3.68 |
| **Glutamate** | 9.39 | 5.06 | 3.64 |
|  | 9.39 | 5.22 | 3.72 |
|  | 9.62 | 5.38 | 3.76 |
|  | 10.36 | 5.47 | 3.85 |
|  | 10.61 | 5.72 | 3.90 |
|  | 10.90 | 6.00 | 3.90 |
|  | 11.21 | 6.00 | 3.95 |
|  | 11.21 | 6.10 | 4.00 |
|  | 11.23 | 6.21 | 4.04 |
|  | 11.53 | 6.42 | 4.09 |
|  | 11.86 | 6.53 | 4.15 |
|  | 11.86 |  |  |
|  | 12.22 |  |  |
