# Supplementary material for: The integrity of synthetic magnesium silicate in charged compounds
Source: Sci Rep. 2021 Dec 9;11:23717. doi: 10.1038/s41598-021-02930-8 (PMC8660837; doi:10.1038/s41598-021-02930-8)
Supplement: Supplementary file 2 — Supplementary Information 2. [file 41598_2021_2930_MOESM2_ESM.docx]

**Supplementary information.** Molecular structures of the charged and neutral compounds used can be found in the supplementary information. SEM images of each solid collected at each pH for each charged compound at additional magnifications (500x, 5,000x, 20,000x, and 50,000x) can be found in the supplementary information. Representative EDS point spectra for each compound at each pH, as well as a table of all the Si/Mg ratios measured, can also be found in the supplementary information.
